# Radiology websites: Fetal imaging

**Published:** 2008-08

**Authors:** IK Indrajit

**Affiliations:** Department of Radiodiagnosis and Imaging, Army Hospital (Research and Referral), Delhi Cantt - 110 010, India

A few useful websites focusing fetal imaging and related topics are reviewed below:

1. **Fetal Medicine** at http://www.fetalmedicine.com/ is an educative portal of Fetal Medicine Foundation, London, UK, created by Prof Kypros Nicolaides. The site has useful sections covering diagnosis of fetal abnormalities on USG scan at 11-14 weeks and at 18-23 week. Useful material on screening for Down syndrome at 11-13 weeks is available at http://www.fetalmedicine.com/f-downs.htm with a downloadable PDF copy. There is an illustrative image atlas of fetus available at http://www.fetalmedicine.ac.uk/scan/img_main.htm. An online version of a textbook on Doppler in Obstetrics, authored by K.H. Nicolaides *et al.* for the Diplomat in Fetal Medicine Series is available at http://www.centrus.com.br/DiplomaFMF/SeriesFMF/doppler/index-doppler.htm.
2. **Fetus.Net** at http://www.thefetus.net/index.php is a ‘non-profit corporation dedicated to promote education about prenatal diagnosis by ultrasound over the web’ and is presented by Philippe Jeanty. There are many absorbing sections (eg, articles, cases, tools, and links) available at http://www.thefetus.net/listing.php?id=1. There are sections on various topics, including embryology, amniotic fluid, cord, Doppler, regional anomalies, hydrops, membranes, multiple gestations, procedures, syndromes, etc. An educative ‘Case of the Week’ section is available at http://www.thefetus.net/listing.php?id=2, where more than 200 cases can be viewed.
3. **Obstetrics Ultrasound Net** at http://www.ob-ultrasound.net/ is a popular site that has won many awards. The site, created by Dr J. S. K. Woo from Hong Kong, is a comprehensive guide to ultrasound for patients, students, medical practitioners, and other healthcare workers. Educative sections available at the site include a) News and Views on Ultrasound Scans at http://www.ob-ultrasound.net/news.html, b) History of Ultrasound at http://www.ob-ultrasound.net/history.html, c) a Fetal Anomaly reference section covering almost 80 fetal anomalies at http://www.ob-ultrasound.net/anomaly.html, and d) Measurements for Down Syndrome at http://www.ob-ultrasound.net/xdown.html
4. **SonoWorld** is a global resource of ultrasound hosted by Medimage World, Philadelphia, and offers ‘free or low-cost educational information to the global ultrasound community.’ The site is the creation of an expert team led by E. Ray, Barry Goldberg, C.R.B. Merritt, and L. Waldroup. A mandatory registration in the opening page is necessary to access the numerous sections; these include Clinical Challenge, Features of the Week and news stories. A pull-down menu leads to numerous cases, images, lectures, articles, and chapters; alternatively, a site map is on offer at http://www.sonoworld.com/Sonoworld/SiteMap/UltrasoundSiteMap.aspx. At the moment, the offered educative material includes ultrasound in partial and complete molar gestation, chapters on fetal measurements, CME in twin pregnancy, and large-for-date and small-for-date fetuses.
5. **Perinatology.com** is an ‘educational resource for perinatologists, referring physicians, and genetic counselors.’ Available at http://www.perinatology.com/index.html, the website is hosted by Focus Information Technology and curated by The San Gabriel Valley perinatal medical group. The material divides the topic into regional sections within which the various anomalies are discussed. Besides, there are pages on and links to Biometry Charts Source, RCOG Protocol Standards and Training, ACR standard for the performance of antepartum obstetrical ultrasound (PDF file). A short concise educative material on Level II Ultrasound-Fetal Structural Abnormalities is available at http://www.perinatology.com/ultrasound.htm.
6. **3D Ultrasound** at http://www.3d-us.org/eng/medic.htm is a multicenter, multiauthor, educative web portal with material contributed from centers across Italy and Portugal, eg, Napoli, Porto, Palermo, and Salerno. The web page has two zones: Expert and Visitor Zones. The expert section has illustrative 3D ultrasound images and movies on fetal brain, heart, fetal skeleton, and fetal biometry.
7. **Greg Devore's Prenatal Diagnosis Homepage** at http://www.fetal.com/ is ‘a website designed to be of assistance to patients and physicians.’ This site has an educative section on screening tests for Down syndrome at http://www.fetal.com/NT%20Screening/00%20Introduction.html. In addition, there are material on 3D and 4D screening on http://www.fetal.com/3D4DScreening/index.html and Intrauterine Growth Restriction at http://www.fetal.com/IUGR/index.html
8. **International Birth Defects Information Systems (IBIS)** at http://www.ibis-birthdefects.org/index.htm is a treasure house of scientific knowledge and information related to birth defects, genetic disorders, and teratology. The mission of the site is amelioration and prevention of birth defects and genetic disorders. Incidentally, the ibis is an Egyptian symbol of wisdom. The site weaves together sections such as IBIS Birth Defects, genetics, cancers, syndromes, malformations, and anomalies along with fact sheets and support groups.
  **Online Multiple Congenital Anomaly / Mental Retardation Syndromes** is a ‘database of structured descriptions of congenital abnormalities associated with mental retardation,’ created in the 1990s by Stanley Jablonski. The site, which is currently not active (and needs updating), is archived at http://www.nlm.nih.gov/archive/20061212/mesh/jablonski/syndrome_toc/toc_a.html. The online database stores data on nearly 700 multiple congenital anomalies syndromes that are associated with mental retardation.
9. **Compendium of Fetal MRI** is available from Beth Israel Deaconess Medical Center, Boston, at http://bidmc.harvard.edu/content/bidmc/departments/radiology/files/fetalatlas/default.htm. This illustrative primer and atlas has separate sections on different body regions. The material has been created by a team comprising of D. Levine, MD, A.S. Smith, L.B.A. Gonsalves, and M.S. Frank MD. A useful set of appendices is available that discuss important issues such as a) Practical Comments: prior to fetal MRI b) Image Quality c) Artifacts, d) MR safety and informed consent, and e) fast MR imaging techniques.

## Endpiece

**Indian College of Radiology and Imaging (ICRI)** (founded in 1974) is the academic wing of the Indian Radiological and Imaging Association. The academic activities of the college are ‘aimed at promotion, practice, and advancement in the science of radiodiagnosis’ and cover all imaging modalities. The website is available at http://icri.co.in/index.htm. It offers sections like Newsletter, Article of the Month, Case of the Month, and an Image Gallery.

**Radiological Society of North America (RSNA)** is a society formed to ‘promote and develop the highest standards of radiology and related sciences through education and research.’ This site, which is frequently updated and periodically reworked to give it a contemporary look, has its opening page at http://www.rsna.org/. This links to sections like Membership, Education, Publications, and Technology, to name a few. In particular, the education portal is packed with academic material, largely sourced from the journal Radiographics. They include **Best Cases from the AFIP** (emphasizing radiologic-pathologic correlation), **AAPM/RSNA Physics Tutorials for Residents**, and **Cases of the Day** (presented at RSNA Scientific Assembly and Annual Meeting since 2001). Special educative sections on **Quality Initiatives, Issues in Electronic Communication/Informatics, Breast Imaging**, and **Cardiac Imaging** are also available. To reach these distinct and useful academic locations, a sitemap available at http://www.rsna.org/sitemap.cfm is immeasurably handy. The technology section offers material on **IHE, MIRC**: Medical Imaging Resource Center, **RadLex** : Radiology Lexicon, **DICOM**, and a Mini Tutorial on Internet.

